# Supplementary material for: Comparative genomics provides new insights into the diversity, physiology, and sexuality of the only industrially exploited tremellomycete: Phaffia rhodozyma
Source: BMC Genomics. 2016 Nov 9;17:901. doi: 10.1186/s12864-016-3244-7 (PMC5103461; doi:10.1186/s12864-016-3244-7)
Supplement: Additional file 6: — List of orphan genes with links to PFAM (related to Additional file 1: Table S1). (ZIP 1428 kb) [file 12864_2016_3244_MOESM6_ESM.zip › BLAST_HTML_FTR/G01395_P.html]

BLAST Search Results


```
BLASTP 2.2.27+


Reference:
Stephen F. Altschul, Thomas L. Madden, Alejandro A. Schäffer,
Jinghui Zhang, Zheng Zhang, Webb Miller, and David J. Lipman (1997),
"Gapped BLAST and PSI-BLAST: a new generation of protein database
search programs", Nucleic Acids Res. 25:3389-3402.


Reference for
composition-based statistics:
Alejandro A. Schäffer, L. Aravind, Thomas L. Madden, Sergei
Shavirin, John L. Spouge, Yuri I. Wolf, Eugene V. Koonin, and
Stephen F. Altschul (2001), "Improving the accuracy of PSI-BLAST
protein database searches with composition-based statistics and
other refinements", Nucleic Acids Res. 29:2994-3005.


Database: nr
           71,551,133 sequences; 26,053,659,533 total letters


Query= G01395_P

Length=107
                                                                      Score     E
Sequences producing significant alignments:                          (Bits)  Value

emb|CED85330.1|  hypothetical protein [Xanthophyllomyces dendrorh...   218    1e-70
gb|ALE52845.1|  hypothetical protein SP60_06305 [Candidatus Thiog...  35.0    3.1  
ref|NP_001143816.1|  uncharacterized protein LOC100276592 [Zea ma...  35.0    7.6  


 >emb|CED85330.1| hypothetical protein [Xanthophyllomyces dendrorhous]
Length=106

 Score =  218 bits (554),  Expect = 1e-70, Method: Compositional matrix adjust.
 Identities = 106/106 (100%), Positives = 106/106 (100%), Gaps = 0/106 (0%)

Query  1    MSLSVKTRVSSPSFRESDQFYDLPSYSATALSIPSYSPDSSSSHIHQRLFAPKRSFGGLI  60
            MSLSVKTRVSSPSFRESDQFYDLPSYSATALSIPSYSPDSSSSHIHQRLFAPKRSFGGLI
Sbjct  1    MSLSVKTRVSSPSFRESDQFYDLPSYSATALSIPSYSPDSSSSHIHQRLFAPKRSFGGLI  60

Query  61   KRLCSLQGRKQARARKEQVEELAEDEKEIAQKMVDQYWDKCISMGY  106
            KRLCSLQGRKQARARKEQVEELAEDEKEIAQKMVDQYWDKCISMGY
Sbjct  61   KRLCSLQGRKQARARKEQVEELAEDEKEIAQKMVDQYWDKCISMGY  106


>gb|ALE52845.1| hypothetical protein SP60_06305 [Candidatus Thioglobus sp. EF1]
Length=115

 Score = 35.0 bits (79),  Expect = 3.1, Method: Compositional matrix adjust.
 Identities = 20/61 (33%), Positives = 31/61 (51%), Gaps = 1/61 (2%)

Query  39  DSSSSHIHQRLFAPKRSFGGL-IKRLCSLQGRKQARARKEQVEELAEDEKEIAQKMVDQY  97
           DS +++ H R F   RS GG  IK    L+G+    +         E+EKEI + + D+Y
Sbjct  40  DSGANYSHNREFYESRSLGGAKIKNFADLKGQINRCSNYYDSAWFPEEEKEIVKYLNDEY  99

Query  98  W  98
           +
Sbjct  100 Y  100


>ref|NP_001143816.1| uncharacterized protein LOC100276592 [Zea mays]
 gb|ACG35647.1| hypothetical protein [Zea mays]
Length=192

 Score = 35.0 bits (79),  Expect = 7.6, Method: Compositional matrix adjust.
 Identities = 22/71 (31%), Positives = 37/71 (52%), Gaps = 2/71 (3%)

Query  27  SATALSIPSYSPDSSSSHIHQRLFAPKRSFGGLIKRLCSLQGRKQARARKEQVEELAEDE  86
           +ATAL +PS +  + +     RL  P+   G L+ R  SLQ  ++ R     V+E  E E
Sbjct  2   AATALLLPSLAAPTDTPRPAHRLLQPQLQRGSLVSR--SLQPPRRRRLAVSAVQETKEGE  59

Query  87  KEIAQKMVDQY  97
            + A ++ ++Y
Sbjct  60  AKTADEITEKY  70


Lambda      K        H        a         alpha
   0.321    0.131    0.381    0.792     4.96 

Gapped
Lambda      K        H        a         alpha    sigma
   0.267   0.0410    0.140     1.90     42.6     43.6 

Effective search space used: 639088976175


  Database: nr
    Posted date:  Sep 23, 2015 12:05 AM
  Number of letters in database: 26,053,659,533
  Number of sequences in database:  71,551,133


Matrix: BLOSUM62
Gap Penalties: Existence: 11, Extension: 1
Neighboring words threshold: 11
Window for multiple hits: 40
```
